# The use of statins for the treatment of depression in patients with acute coronary syndrome

**DOI:** 10.1038/tp.2015.116

**Published:** 2015-08-18

**Authors:** S W Kim, K Y Bae, J M Kim, I S Shin, Y J Hong, Y Ahn, M H Jeong, M Berk, J S Yoon

**Affiliations:** 1 Department of Psychiatry, Chonnam National University Medical School, Gwangju, Korea; 2 Department of Cardiology, Chonnam National University Medical School, Gwangju, Korea; 3 IMPACT Strategic Research Centre, School of Medicine, Deakin University, Geelong, VIC, Australia; 4 Department of Psychiatry, The University of Melbourne, Parkville, VIC, Australia

## Abstract

This study aimed to investigate the effect of statins for the treatment of depression in individuals with acute coronary syndrome (ACS). We used 1-year follow-up data of a 24-week double-blind, placebo-controlled trial of escitalopram and a naturalistic prospective observational cohort study. Of 446 participants with comorbid depressive disorders and ACS at baseline, 300 participated in a randomised escitalopram trial and the remaining 146 participated in a naturalistic observational study. The participants in the two studies were approached for a 1-year follow-up investigation. Treatment response rates, defined as a ⩾50% reduction in the Hamilton Depression Rating Scale (HAM-D) and Beck Depression Inventory (BDI) scores, were used as the outcome variables. In the escitalopram trial, both HAM-D and BDI response rates were highest in patients taking escitalopram and statins together and lowest in patients receiving neither medication. Logistic regression analyses revealed that statin use was significantly associated with higher response rates on both the HAM-D and BDI at 1 year, whereas no such associations were found for escitalopram. In the naturalistic observational study, the response rates at 1 year did not differ significantly by statin use. Instead, the HAM-D response rate was significantly higher in patients taking lipophilic statins than in those who did not. In conclusion, statins may be effective for the treatment of depression independent of medical status and escitalopram use, and they may potentiate the antidepressant action of serotonergic antidepressants in patients with ACS.

## Introduction

Statins (3-hydroxy-3-methylglutaryl coenzyme A reductase inhibitors) are widely used for primary and secondary prevention of cardiovascular disease by treating hypercholesterolaemia. Low mood and suicide have been reported as possible side effects of cholesterol-lowering treatment with statins.^1^ The association between lower cholesterol and depression/suicide was been hypothesised as being related to reduced serotonin in the brain.^2^ Nevertheless, a more recent meta-analysis^3^ of randomised controlled trials demonstrated no adverse effects of statins on psychological outcomes, and a systemic review and meta-analysis^4^ of observational studies suggested that statin use might be associated with lower risk of depression. Furthermore, a few studies suggested that statins have an antidepressant effect^5^ independent of cholesterol-lowering effects.^6^ To date, only two small randomised controlled trials of statins has been published, suggesting antidepressant properties adjunctive to serotonin reuptake inhibitors in the general population with major depressive disorder.^7, 8^

The inflammatory hypothesis of depression is now well established,^9, 10^ and statins have known effects on inflammatory cytokines, and also reduce oxidative stress markers.^11^ Statins exert anti-inflammatory activity, including reducing C-reactive protein concentration in both healthy individuals and those with stable vascular disease.^12^ In patients who experience an acute cardiac event, statins induce a rapid reduction in tumour necrosis factor alpha and interferon gamma production in stimulated T-lymphocytes, and inhibit the T helper cell (Th-1) immune response.^13^ In human hepatocytes, statins reduce interleukin-6-induced expression of C-reactive protein, suggesting a hepatic source of its anti-inflammatory effects.^14^ Furthermore, the low-density lipoprotein cholesterol-lowering effect of statins may be associated with anti-inflammatory effects, because low-density lipoprotein cholesterol itself strongly promotes inflammation.^15^ These anti-inflammatory effects and antioxidant effects of statins are thought to be associated with antidepressant effects.^11, 16^

However, studies demonstrating the effects of statins on depression in individuals with acute coronary syndrome (ACS; that is, any condition brought on by sudden reduced blood flow to the heart, including myocardial infarction or unstable angina) are limited. In addition, to our knowledge, no previous reported study exists that identifies the interaction between statins and serotonin reuptake inhibitors on antidepressant effects in individuals with ACS. This study aimed to investigate the effects of statins on depression in a Korean population with ACS. We used 1 year follow-up data of a 24-week double-blind, placebo-controlled trial of escitalopram for treatment of depressive disorder in patients with ACS (Escitalopram for DEPression in ACS, EsDEPACS)^17^ and a naturalistic prospective observational cohort study (Korean DEPression in ACS, K-DEPACS).^18^

## Materials and methods

### Study design and participants

The present study included subjects with comorbid ACS and depression at baseline in the K-DEPACS study. The K-DEPACS study is a naturalistic prospective cohort study that began in 2006. Comprehensive study details have been published previously.^17, 18^ Participants were recruited from those patients 2–14 weeks after an ACS episode that had been confirmed by various examinations and that resulted in hospitalisation (*n*=4809) in the central coordinating centre of the Korea Acute Myocardial Infarction Registry.

Patients who met the eligibility criteria and agreed to participate (*n*=1152) were screened for depressive symptoms using the Beck Depression Inventory (BDI).^19^ Those with a score indicative of depression (>10) underwent a structured diagnostic evaluation conducted by research psychiatrists using the Mini-International Neuropsychiatric Interview,^20^ which categorises patients as having depressive disorder. Of them, 446 (38.7%) who met the Diagnostic and Statistical Manual of Mental Disorders, Fourth Edition (DSM-IV)^21^ criteria for major or minor depressive disorder were subjects in the present study. Of these, 300 (67.3%) participated in the EsDEPACS trial, which was a 24-week, double-blind randomised controlled trial of escitalopram (ClinicalTrials.gov registry number: NCT00419471). The remaining 146 individuals with depression did not participate in the clinical trial and only received standard medical treatment (K-DEPACS cohort). All participants in the two studies were approached for 1-year follow-up investigation. Written informed consent was obtained from all participants. Both studies were approved by the Chonnam National University Hospital Institutional Review Board.

**Figure 1:**
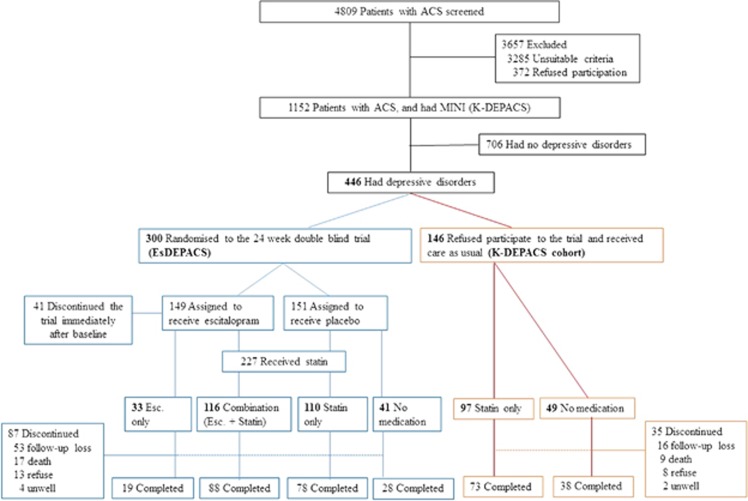
Flow diagram for recruitment and grouping according to treatment status. ACS, acute coronary syndrome; Esc., escitalopram; EsDEPACS, the Escitalopram for Depression in Acute Coronary Syndrome trial; K-DEPACS, the Korean Depression in Acute Coronary Syndrome study.

In total, 300 patients from the EsDEPACS trial were randomised to either escitalopram (*n*=149) or placebo (*n*=151). Statins were prescribed according to medical indications as determined by the treating cardiologists (*n*=226). To identify the effects of statins and interactions between statins and escitalopram, the 300 participants of the EsDEPACS trial were divided into four groups according to escitalopram and statin use, as follows: use of both escitalopram and statins (Combination: *n*=116, 38.7%), Escitalopram only (subjects who were administered escitalopram but were not prescribed a statin, *n*=33, 11.0%), Statin only (subjects who were prescribed a statin but were not administered escitalopram, *n*=110, 36.7%) and No medication (subjects who were not administered escitalopram or a statin and received standard medical treatment only, *n*=41, 13.7%) (Figure 1). A total of 213 patients (71.0%) completed a 1-year follow-up evaluation, with no significant differences in follow-up rates among the four groups (Combination 75.9%, Escitalopram only 57.6%, Statin only 70.9%, No medication 68.3% *P*=0.225).

Among 146 participants who had depression in the K-DEPACS cohort study, 97 (66.4%) patients received a statin (statin group) and 49 (33.6%) patients received standard medical treatment only (no-medication group). A total of 111 subjects (76.0%) completed a 1-year follow-up evaluation, with no significant differences in follow-up rates among the two groups (Statin 75.3% and No medication 77.6%, *P*=0.759).

### Measures

The primary outcome measure in the EsDEPACS trial and the K-DEPACS cohort study was the 17-item Hamilton Depression Rating Scale (HAM-D).^22^ The BDI was selected as a secondary outcome measure in the present study.

Various cardiac risk factors and medical status were investigated. Fasting total cholesterol level was assayed; hypercholesterolaemia was diagnosed when the cholesterol level was >200 mg dl^−1^ or if patients had a history of ongoing hyperlipidemia treatment. Body mass index was calculated, and participants were categorised as obese in cases of body mass index >25 kg/m^2^. To evaluate current cardiac status, diagnoses of myocardial infarction or unstable angina were made. ACS severity was estimated (range: I–IV) using the Killip classification.^23^ Left ventricular ejection fraction was estimated using echocardiography. Serum troponin I was measured as a cardiac biomarker.

### Study procedures

The details of the EsDEPACS trial have been published, in which superior effects of escitalopram to placebo were found.^17^ In brief, subjects were randomised to the escitalopram and placebo conditions in a 1:1 ratio following computer-generated randomisation codes. Flexible doses of study medications ranging from 5 to 20 mg were administered for 24 weeks by a blinded psychiatrist. The mean (s.d.) doses at the last visit were 7.6 (3.7) mg for the escitalopram group and 8.5 (3.9) mg for the placebo group, and the mean (s.d.) treatment durations were 19.8 (3.2) and 19.7 (3.3) weeks, respectively.

Concomitant medications, such as any other antidepressant, psychostimulant, antipsychotic or anticholinergic agents, were not permitted. In the K-DEPACS cohort, standard medical treatments were provided to all subjects by the study cardiologists based on international guidelines for the management of ACS. Outcome measures were blindly assessed following the prescription of statins as well as escitalopram.

### Statistical analyses

The main analyses were performed on an intent-to-treat basis, including all patients who were randomly assigned to treatment. Demographic and clinical characteristics at baseline were compared according to treatment condition using *χ*^2^-tests, analysis of variance or independent *t*-tests. Treatment response rates at 1 year, defined as a ⩾50% reduction in the HAM-D or BDI baseline score, were used as primary outcome variables. Response rates were also calculated at 24 weeks in the EsDEPACS trial. Response rates among the groups were compared using *χ*^2^-tests. Proportions of participants who no longer met DSM-IV criteria for major or minor depressive disorder at 1 year among the groups were also compared using *χ*^2^-tests. Repeated-measures analysis of variance treating time as the within-subject factor and treatment group as the between-subjects factor demonstrated changes in HAM-D and BDI scores over time in the EsDEPACS trial after adjusting for baseline scores. Logistic regression models were used to investigate independent effects of escitalopram and statins on treatment response after adjusting for baseline score of the depression measure, demographic and clinical characteristics that were not equally distributed among the groups (*P*<0.1) in unadjusted analyses, key cardiac parameters (myocardial infarction, Killip class and total cholesterol level) and for each drug (escitalopram and statins). In the EsDEPACS trial, 41 subjects randomly assigned to the escitalopram group discontinued the trial immediately after their baseline visit. Therefore, exploratory regression analyses of the 1-year follow-up evaluations excluded these subjects, who may have shown no medication effects. Another exploratory analyses were conducted to compare differential effects according to statin type: lipophilic statins (simvastatin, atorvastatin, lovastatin and pitavastatin) versus hydrophilic statins (rosuvastatin and pravastatin).^24^ A *P*-value <0.05 (two tailed) was taken to indicate statistical significance, and statistical analyses were performed using the SPSS software (ver. 21.0; SPSS, Chicago, IL, USA).

## Results

Baseline characteristics by treatment status are compared in Table 1. No significant differences in demographic or clinical characteristics, except for the prevalence of obesity, were found among the four groups in the EsDEPACS trial. Prevalence of hypertension and serum cholesterol levels tended to be different among the groups, but the differences were not statistically significant (*P*=0.091 and 0.090, respectively). In the K-DEPACS cohort, serum levels of troponin-I and total cholesterol were significantly higher in the statin-only group than in the no-medication group.

### Analysis of the EsDEPACS trial

**Figure 2:**
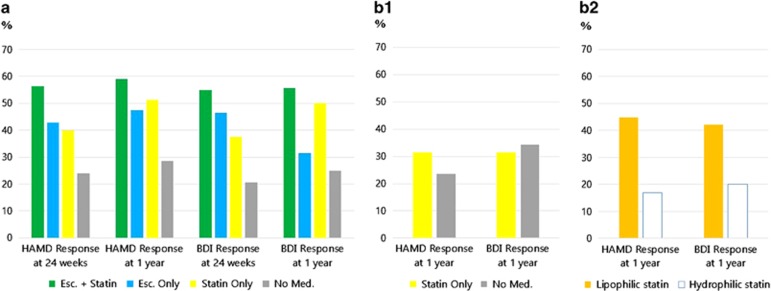
Response rates at 24 weeks and 1 year according to treatment status in the EsDEPACS trial and K-DEPACS cohort. (**a**) EsDEPACS trial sample. HAM-D and BDI response rates at both 24 weeks and 1 year differed significantly among treatment groups (*P*=0.018, 0.045, 0.008 and 0.017, respectively). (**b1**) K-DEPACS cohort sample. HAM-D and BDI response rates at 1 year did not differ significantly according to statin use (*P*=0.388 and 0.773, respectively). (**b2**) K-DEPACS cohort sample. HAM-D and BDI response rates at 1 year were significantly higher in patients who took lipophilic statins than in those who took hydrophilic statins (*P*=0.011 and 0.042, respectively). BDI, Beck Depression Inventory; Esc., escitalopram; EsDEPACS, the Escitalopram for Depression in Acute Coronary Syndrome trial; HAM-D, Hamilton Depression Rating Scale; K-DEPACS, the Korean Depression in Acute Coronary Syndrome study; Med., medication.

In the EsDEPACS trial, HAM-D and BDI response rates at both 24 weeks and 1 year differed significantly among treatment groups (Figure 2). Both HAM-D and BDI response rates at 24 weeks and 1 year were highest in patients taking escitalopram and statins together and lowest in patients receiving neither medication. Proportion of participants who no longer met DSM-IV criteria for major or minor depressive disorder at 1 year was also highest in the combined treatment group, but differences among treatment groups were not statistically significant (Combination 65.9%, Escitalopram only 52.6%, Statin only 50.0%, No medication 42.9% *χ*^2^**=**6.691, *P*=0.082).

In the logistic regression, statin use was significantly associated with higher response rates on both the HAM-D and BDI at 1 year, whereas escitalopram use was not associated with the response rates on the HAM-D and BDI at 1 year after adjusting for significant covariates (Table 2). However, escitalopram was significantly associated with higher response rates on both the HAM-D and BDI at 24 weeks, whereas statin use was not significantly associated with these outcomes at 24 weeks. When we excluded from the analysis subjects who were randomly assigned to receive escitalopram but who discontinue the clinical trial after the first visit, both statin and escitalopram use were found to be significantly associated with higher response rates on the HAM-D and BDI at 1 year.

**Figure 3:**
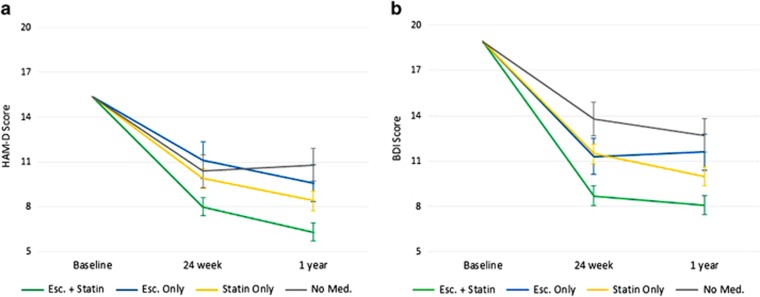
Adjusted mean Hamilton Rating Scale for Depression (HAM-D) and Beck Depression Inventory (BDI) scores over time in the EsDEPACS trial. (**a**) Statistics for group by time interaction after adjustment for baseline HAM-D score are F=2.839 and *P*=0.010. (**b**) Statistics for group by time interaction after adjustment for baseline BDI score are F=2.357 and *P*=0.030. Esc., escitalopram; EsDEPACS, the Escitalopram for Depression in Acute Coronary Syndrome trial; Med., medication.

Figure 3 illustrates changes in HAM-D and BDI scores over time according to group. There was a significant time × group interaction for both the HAM-D and BDI scores (*P*=0.010 and 0.030, respectively). Similar to the response-rate analyses, HAM-D and BDI scores were most improved in patients taking escitalopram and statins together.

Escitalopram as a study medication was not provided to subjects after the 24-week trial period. Escitalopram was being taken by eight patients at the 1-year follow-up point on the basis of clinician's judgment. However, antidepressant use at 1 year did not differ significantly among the groups. Furthermore, the results were not changed substantially when the same analyses were repeated after excluding these participants.

Exploratory analyses were conducted to compare the efficacy of lipophilic statins (*n*=141) and hydrophilic statins (*n*=85), as the former theoretically have greater brain permeability. In the EsDEPACS trial, there was no significant difference in HAM-D or BDI response rates between these two groups (all *P*>0.1).

Safety results were reported in our previous study.^17^ In brief, dizziness was more frequently reported in patients taking escitalopram compared with those who did not take it (13.9 vs 4.6%, *P*=0.018). However, there were no significant differences with respect to any other adverse events according to use of escitalopram or statin.

### Analysis of the K-DEPACS cohort

HAM-D and BDI response rates at 1 year did not differ significantly according to statin use (Figure 2). Proportions of participants who no longer met DSM-IV criteria for major or minor depressive disorder at 1 year were not significantly different between the two groups (Statin 52.1% and No medication 44.7%, *χ*^2^=0.535, *P*=0.464). Logistic regression analysis also demonstrated no significant association between statin use with HAM-D or BDI response rates (Table 3). Antidepressants were being taken by only two patients, who were in the no-medication group, at the 1-year follow-up point. When the same analyses were repeated after excluding them, the results were not substantially changed.

In the K-DEPACS cohort, HAM-D and BDI response rates were significantly higher in patients who took lipophilic statins (*n*=52) than in those who took hydrophilic statins (*n*=45) (44.7 vs 17.1% and 42.1 vs 20.0%, respectively). No demographic or clinical characteristic differed significantly between the lipophilic and hydrophilic statins groups. Patients receiving hydrophilic statins were more likely to have diabetes (26.7% vs 11.5%, *P*=0.056). Logistic regression analysis was conducted to investigate the independent effects of lipophilic statins compared with hydrophilic statins after adjusting for baseline score of depression measure and clinical status (diabetes, total cholesterol level, type of ACS and Killip classification) (Table 3). Patients receiving lipophilic statins were significantly more likely to achieve HAM-D response at 1 year compared with those receiving hydrophilic statins, after adjusting for these variables. Lipophilic statin use was also associated with higher BDI response at 1 year compared with hydrophilic statin use, but it was only a trend in a multivariate analysis (*P*=0.054). When subjects were divided into two groups according to use of lipophilic statins, patients who took lipophilic statins (*n*=52) were significantly more likely to achieve HAM-D response at 1 year than those who did not (no-medication and hydrophilic statin group, *n*=94).

## Discussion

Although there are a few studies suggesting beneficial effects of statins on depression in the general population, studies demonstrating antidepressant effect of statins in individuals with ACS are limited. The present study showed improvement in depressive symptoms associated with statin use in individuals with ACS and depressive disorder. These effects of statins at the 1-year follow-up point were independent of medical status and escitalopram use. However, in both subjective and objective measures, response rates in the group receiving combined statin and escitalopram treatment were highest among the four groups. To our knowledge, this is the first reported study to demonstrate an antidepressant effect of statins and an interaction between statins and escitalopram in individuals with ACS and depression.

The pathophysiology and aetiology of depression are heterogeneous and complex. Monoamine deficiency was hypothesised to be the main cause of depression, and medications to compensate for this deficiency have been most widely used for the treatment of depression. However, the monoaminergic theory of depression has failed to deliver novel agents beyond the limited treatment options currently available.^25^ Inflammation, oxidative stress and stress reactions have also recently been suggested operative neurobiological pathways in depression.^26^ A growing body of literature illustrates the presence of increased inflammatory biomarkers, including inflammatory cytokines, in patients with depression.^27^ However, medications mainly targeting inflammatory and oxidative pathways have not been widely used in clinical settings. Our results are in accordance with previous novel clinical trials suggesting antidepressant effects of anti-inflammatory agents such as celecoxib, pioglitazone and *N*-acetylcysteine.^28, 29, 30, 31^ Furthermore, findings of the highest response rate in the combination of escitalopram and statin group (escitalopram use for 24 weeks and statin use for 1 year) suggests that statins may potentiate the efficacy of serotonergic antidepressants. This result is in accordance with a few recent controlled trials showing that augmentation of statins (lovastatin and atorvastatin) to serotonin reuptake inhibitors (fluoxetine and citalopram) may be effective for treating depression in the general population with major depressive disorder.^7, 8^

In the intention-to-treat population of this study, the effect of escitalopram treatment on depressive symptoms was not observed at the 1-year follow-up point. This might be attributed to high proportions of subjects who had discontinued clinical trial after baseline visit. In fact, effect of escitalopram was seen at 1-year follow-up when the analysis was conducted in subjects who received escitalopram for at least 4 weeks. Given that almost all subjects had discontinued escitalopram at 24 weeks when the clinical trial was completed, and given that the mean duration of escitalopram treatment was about 20 weeks in the EsDEPACS trial, it appears that the effects of escitalopram use persist over a long period of time in patients with ACS. Our findings also suggest potential maintenance effects of the statin on depression. In some populations with ACS, escitalopram use may be limited due to its potential to increase the risk of bleeding or QTc interval prolongation.^32, 33^ In this case, statins may be a novel alternative to treat depression or maintain the antidepressant effects of a serotonin reuptake inhibitor.

In contrast to the results of clinical trial sample, statin use was not effective for the treatment of depression in the naturalistic observational study sample. This difference in result may be attributed to interactions of statin use with escitalopram as well as the placebo, which can biologically modulate the release of opioids, dopamine, serotonin and immune mediators.^34^ In addition, the clinical trial sample regularly met a psychiatrist for 24 weeks but subjects in the naturalistic observational study did not.

In addition, residual confounding in non-randomised trials is an important factor. The factors that drive statin prescription are known risk factors for depression. These data, as well as multiple other studies, suggest that obesity is a risk factor for depression. There is also extensive data that a poor-quality diet is a risk factor for depression,^35^ as is physical inactivity.^36^ These factors drive statin prescription, together with other risk factors for cardiovascular disease such as smoking, which itself is a risk factor for depression.^37^ As a consequence, one could reasonably expect participants prescribed statins to actually be at a considerably higher risk of depression. As a consequence, it is entirely feasible that the null finding in the non-randomised study, at least in part, reflects these factors. In addition, because of this residual confounding, non-randomised trials are likely to underestimate the antidepressant effects of statins.

There are two distinct types of statin, when classified by their lipophilicity. Lipophilic statins and hydrophilic statins exert differential effects on the human body.^24, 38, 39, 40^ In the naturalistic observational study sample, the response rates with hydrophilic statins were significantly lower than those of lipophilic statins, which were similar to those with statins in the clinical trial sample. The relatively high response rates with hydrophilic statins used in the clinical trial sample, which resulted in the absence of a difference in effects according to the type of statins, may be also attributable to interactions with escitalopram and the placebo effect. The reason for the greater effect of lipophilic statins is unclear. The degree of lipophilicity required to pass the blood–brain barrier may be associated with direct effects on the brain. Specifically, lipophilic statins are more likely to cross the blood–brain barrier than are hydrophilic statins.^41^ In addition, lipophilic statins enter cells by passive diffusion and are thus widely distributed in different tissues, whereas hydrophilic statins are more liver-specific and are taken up via carrier-mediated mechanisms, thus reducing their ability to exert non-lipid effects in extrahepatic tissues.^41^

This study has some limitations. First, we should be cautious of generalising the antidepressant effects of statins to the general population, because this study was conducted only in individuals with ACS. Second, psychological treatment effects were not fully controlled in the clinical trial when patients met a psychiatrist who prescribed study medications. Third, the severity of our participants' depression was relatively low compared with that of patients in other clinical trials of depression; further study is warranted to investigate whether statins are effective in populations with severe depression. Fourth, the escitalopram-only group contained a relatively small number of subjects compared with the other groups. Furthermore, the study design combined a randomised controlled trial with a naturalistic observational study. Therefore, statins were administered to the subjects by cardiologists without randomisation, whereas administration of escitalopram was randomised; as mentioned above, this residual confounding may underestimate the effects of statins. However, depression assessments were conducted blind, independent of statin use. Further study should include randomisation of both escitalopram and statin to improve statistical power and remove residual confounding.

In conclusion, the results of this study suggest that statins may be effective for the treatment of depression independent of medical status and escitalopram use, and they may potentiate the antidepressant action of serotonergic antidepressants in patients with ACS. In some patients with ACS and depressive disorder who are not tolerant to antidepressants, statins may be a novel alternative.

## Figures and Tables

**Table 1:** Baseline characteristics by treatment status in all depressive patients after ACS (*n*=446)

	*EsDEPACS trial (*N=*300)*					*K-DEPACS cohort (*N=*146)*		
	*Esc.+Statin*	*Esc. only*	*Statin only*	*Placebo only*	P*-value*	*Statin use*	*Standard treatment*	P*-value*
	N *(%)*	N *(%)*	N *(%)*	N *(%)*				
	*116 (38.7)*	*33 (11.0)*	*110 (36.7)*	*41 (13.7)*		*97 (66.4)*	*49 (33.6)*	
*Demographic characteristics*								
Age, mean (s.d.) years	60.3 (10.8)	58.9 (12.9)	60.4 (10.8)	59.3 (10.0)	0.855	57.8 (11.8)	59.3 (11.2)	0.458
Gender, *N* (%) men	66 (56.9)	22 (66.7)	63 (57.3)	30 (73.2)	0.223	59 (60.8)	25 (51.0)	0.258
Education, mean (s.d.) years	9.2 (4.3)	10.1 (3.7)	9.0 (4.2)	9.6 (3.8)	0.616	8.9 (5.4)	9.0 (4.5)	0.891

*Depression characteristics*								
HAM-D, mean (s.d.) score	15.6 (4.7)	16.8 (5.1)	15.9 (5.0)	14.9 (4.1)	0.374	10.7 (3.5)	11.3 (3.4)	0.327
BDI, mean (s.d.) score	18.5 (8.3)	20.0 (8.5)	19.2 (7.8)	19.1 (7.3)	0.782	14.0 (7.2)	14.5 (5.5)	0.650

*DSM-IV diagnosis*, N *(%)*								
Major depressive disorder	63 (54.3)	22 (66.7)	59 (53.6)	25 (61.0)	0.511	18 (18.6)	15 (30.6)	0.100

*Cardiac risk factors*, N *(%)*								
Hypertension	64 (55.2)	26 (78.8)	70 (63.6)	24 (58.5)	0.091	43 (44.3)	25 (51.0)	0.444
Diabetes mellitus	33 (28.4)	11 (33.3)	31 (28.2)	10 (24.4)	0.868	18 (18.6)	10 (20.4)	0.788
Hypercholesterolaemia	61 (52.6)	12 (36.4)	54 (49.1)	17 (41.5)	0.318	57 (58.8)	21 (42.9)	0.069
Obesity	46 (39.7)	13 (39.4)	55 (50.0)	10 (24.4)	**0.038**	42 (43.3)	18 (36.7)	0.447
Current smoker	33 (28.4)	10 (30.3)	28 (25.5)	14 (34.1)	0.754	34 (35.1)	10 (20.4)	0.069

*Current cardiac status*								
ACS diagnosis, *N* (%)								
Myocardial infarction	77 (66.4)	15 (45.5)	70 (63.6)	22 (53.7)	0.111	31 (32.0)	21 (42.9)	0.194
Killip class >1, *N* (%)	20 (17.2)	4 (12.1)	28 (25.5)	7 (17.1)	0.248	18 (18.6)	10 (20.4)	0.788
LVEF, mean (s.d.) %	60.7 (10.6)	59.4 (12.3)	60.4 (11.4)	62.3 (12.0)	0.561	59.1 (12.2)	62.0 (13.1)	0.190
Troponin I, mean (s.d.) mg dl^−1^	10.0 (9.0)	8.6 (6.4)	10.2 (8.6)	8.2 (5.7)	0.481	11.3 (17.4)	6.2 (12.2)	**0.043**
CK-MB, mean (s.d.) mg dl^−1^	17.5 (23.0)	16.1 (18.3)	17.0 (21.7)	14.5 (18.3)	0.888	20.1 (47.4)	15.8 (29.8)	0.570
T-cholesterol	184.3 (38.0)	171.3 (42.6)	188.6 (39.8)	175.4 (43.8)	0.090	195.4 (38.4)	180.6 (47.8)	**0.045**
CRP	1.1 (0.9)	1.2 (1.1)	1.4 (3.2)	0.7 (0.3)	0.303	1.0 (1.1)	1.2 (2.7)	0.480

Abbreviations: ACS, acute coronary syndrome; BDI, Beck Depression Inventory; CK-MB, creatine kinase-MB; CRP, C-reactive protein; DSM-IV, Diagnostic and Statistical Manual of Mental Disorders 4th edition; Esc., escitalopram; EsDEPACS, the Escitalopram for Depression in Acute Coronary Syndrome trial; HAM-D, Hamilton Depression Rating Scale; K-DEPACS, the Korean Depression in Acute Coronary Syndrome study; LVEF, left ventricular ejection fraction; T-cholesterol, total cholesterol.

Values in bold show statistical significance (*P*-value=0.05).

**Table 2:** Logistic regression analysis of response status by statin and escitalopram use in the EsDEPACS trial

	*HAM-D response (1 year)*		*BDI response (1 year)*		*HAM-D response (24 weeks)*		*BDI response (24 weeks)*	
	*OR (95% CI)*	P*-value*	*OR (95% CI)*	P*-value*	*OR (95% CI)*	P*-value*	*OR (95% CI)*	P*-value*
	*ITT set*								
Statin use	2.23 (1.11–4.51)	**0.025**	2.82 (1.35–5.90)	**0.006**	1.81 (0.94–3.50)	0.078	1.74 (0.89–3.41)	0.108	
Escitalopram use	1.57 (0.90–2.75)	0.111	1.40 (0.79–2.49)	0.250	2.18 (1.23–3.85)	**0.007**	2.55 (1.43–4.55)	**0.002**	
									
	*Efficacy set*								
Statin use	2.84 (1.32–5.87)	**0.005**	3.32 (1.57–7.05)	**0.002**	NA		NA		
Escitalopram use	2.35 (1.29–4.31)	**0.006**	1.88 (1.02–3.47)	**0.044**					

Abbreviations: BDI, Beck Depression Inventory; CI, confidence interval; EsDEPACS, the Escitalopram for Depression in Acute Coronary Syndrome trial; HAM-D, Hamilton Depression Rating Scale; ITT, intention-to-treat; NA, not applicable; OR, odds ratio.

Adjusted for baseline score of depressive measure, obesity, hypertension, total cholesterol level, myocardial infarction diagnosis, Killip class and each use of statin and escitalopram.

The efficacy set group excluded subjects who were randomly assigned to the escitalopram arm but discontinued the trial immediately after the baseline visit.

Values in bold show statistical significance (*P*-value=0.05).

**Table 3:** Logistic regression analysis of response status by statin use in the K-DEPACS cohort

	*HAM-D response (1 year)*		*BDI response (1 year)*	
	*OR (95% CI)*	P*-value*	*OR (95% CI)*	P*-value*
Statin use vs no use	1.19 (0.45–3.18)	0.726	0.89 (0.36–2.22)	0.798
Lipophilic statin use vs hydrophilic statin use	3.91 (1.21–12.59)	**0.022**	2.97 (0.98–9.00)	0.054
Lipophilic statin use vs all others	2.91 (1.21–6.99)	**0.017**	2.00 (0.86–4.66)	0.108

Abbreviations: BDI, Beck Depression Inventory; CI, confidence interval; HAM-D, Hamilton Depression Rating Scale; K-DEPACS, the Korean Depression in Acute Coronary Syndrome study; OR, odds ratio.

Adjusted for baseline score of depression measure, diabetes, total cholesterol level, type of acute coronary syndrome and Killip classification.

Values in bold show statistical significance (*P*-value=0.05).

## Notes

SWK has received grant/research support from Mitsubishi Tanabe, Janssen, Otsuka and National Research Foundation of Korea Grant. JMK has received grant/research support from Ministry of Health and Welfare, Republic of Korea, Lundbeck, Eli Lilly, and Otsuka. The remaining authors declare no conflict of interest.
